# Application of machine learning algorithms in building health diagnostics: predictive analytics evaluating indoor air quality and sick building syndrome in educational settings

**DOI:** 10.7717/peerj.20964

**Published:** 2026-07-09

**Authors:** Syazwan Aizat Ismail, Haslinda Mohamed Kamar, Nazri Kamsah, Kuang Hock Lim, Nur Azzalia Kamaruzaman, Muhammad Iftishah Ramdan, Björn Crüts, Muaz Mohd Zaini Makhtar, Mohd Rafatullah, Aseem Vashisht

**Affiliations:** 1 National Poison Centre, Universiti Sains Malaysia, Gelugor, Pulau Pinang, Malaysia; 2 Faculty of Mechanical Engineering, Universiti Teknologi Malaysia, Skudai, Johor Bahru, Malaysia; 3 National Institutes of Health (NIH), Ministry of Health Malaysia, Setia Alam, Selangor, Malaysia; 4 School of Mechanical Engineering, Universiti Sains Malaysia, Nibong Tebal, Pulau Pinang, Malaysia; 5 Neuro.online, Heerlen, Limburg, Netherlands; 6 Institute of Nano Optoelectronics Research and Technology, Universiti Sains Malaysia, Gelugor, Pulau Pinang, Malaysia; 7 School of Industrial Technology, Universiti Sains Malaysia, Gelugor, Pulau Pinang, Malaysia; 8 University Centre for Research and Development, Chandigarh University, Mohali, Punjab, India

**Keywords:** Internet of Things, Occupational Safety and Health, TinyML, Artificial Neural Networks, Total Volatile Organic Compounds (TVOC), Occupational Hygiene, Health Risk Assessment, Predictive Analytics, Edge Computing, Indoor Environmental Quality (IEQ)

## Abstract

Our research aimed to develop and validate a predictive analytics model for diagnosing sick building syndrome (SBS) in learners. We achieved this by gathering and analyzing epidemiological and exposure assessment data. The current assessment involved the use of the modified MM040NA SBS questionnaire and checklist from Indoor Air Quality (IAQ) Industry Code of Practice (IAQ-ICOP) from Department Occupational Safety and Health (DOSH), Malaysia, with participants scoring of their answers, recording and scoring of their simultaneous self-reported and physician-ascertained health complaints. At the same time, IAQ assessments were collected at the location of each participant with the use of occupational hygiene techniques. Several predictive analytics algorithms, namely Neural Network, Logistic Regression, Classification Tree, Random Forest, and Support Vector Machine, were used to train and test the collected data set. The Neural Network model rendered the most effective classification accuracy, reaching 73.8%. Validation also showed that multiple IAQ parameters were strongly associated with health complaints, especially in mechanically ventilated environments. Variable importance analysis identified Formaldehyde and Total Volatile Organic Compounds (TVOCs) as the top predictors for health complaints, highlighting their critical role in indoor environmental quality. Taken together, the results confirm the effectiveness of neural network-based predictive analytics in correctly diagnosing sick building syndrome (SBS) and related health complaints on limited IAQ data and thereby improving the ability to assess during the early stages.

## Introduction

The management of indoor air quality (IAQ) presents a critical challenge for professionals involved in the design, engineering, and maintenance of buildings within tropical regions, including nations such as Singapore, Thailand, and Malaysia (Department of Occupational Safety Health, 2010). These equatorial locations inherently experience elevated temperatures and humidity, which intensify the potential for issues related to thermal comfort and moisture accumulation, thereby compromising the overall indoor atmospheric conditions (Department of Occupational Safety Health, 2010). Recognizing these unique environmental considerations, the Department of Occupational Safety and Health (DOSH) under Malaysia’s Ministry of Human Resources implemented updated guidelines in 2010, aiming to foster a proactive approach to IAQ risk evaluation within organizational settings. A salutary indoor environment hinges on excellent air quality. Conversely, deficiencies in IAQ are consistently linked to a broad spectrum of adverse health outcomes, ranging from acute irritations to chronic conditions. These include, but are not limited to, respiratory distress, ocular irritation, sinusitis, allergic responses, and more severe infections such as pneumonia and bronchitis (Zamani, Jalaludin & Shaharom, 2013; Ismail, Deros & Leman, 2010). Such atmospheric challenges often originate from sub-optimal performance of mechanical ventilation and air conditioning (MVAC) systems or individual air-cooled units (Zamani, Jalaludin & Shaharom, 2013; Ismail, Deros & Leman, 2010). Contributing factors frequently include insufficient maintenance practices, inadequate regulation of internal environmental parameters, and a lack of established protocols for occupants to actively manage indoor environmental risks.

Key sources of indoor atmospheric contamination include environmental tobacco smoke (ETS), which has been identified in local studies as a significant detrimental factor impacting workplace air quality (Zamani, Jalaludin & Shaharom, 2013). Beyond tobacco smoke, various other chemical agents, such as volatile organic compounds (VOCs) emanating from solvents, ozone produced by office equipment like photocopiers, and formaldehyde released from furniture, can directly impair the air supplied *via* ventilation systems (Department of Occupational Safety Health, 2010; Zamani, Jalaludin & Shaharom, 2013; Ismail, Deros & Leman, 2010; Azid et al., 2015; Ahmad & Hassim, 2015). To mitigate these risks and safeguard the health, well-being, and productivity of employees and other building users, the Industry Code of Practice-Indoor Air Quality (ICOP-IAQ) standard was introduced. This framework outlines the responsibilities of employers and self-employed individuals to their workforce, aligning with Section 15 of the Occupational Safety and Health Act 1994 (OSHA-Malaysia), ensuring active risk control within all operational environments. The scope of these protective measures extends comprehensively to all building occupants, including students and visitors. Given that many airborne contaminants are undetectable without specialized equipment, they often go unnoticed until their negative effects on sensitive individuals become apparent, potentially leading to widespread health complaints or incidents of sick building syndrome. Therefore, preventing future health burdens necessitates the deployment of dependable and foresighted methodologies for quantifying indoor air pollution hazards, thereby minimizing subjective assessment. This involves establishing clear links between quantifiable epidemiological indicators and occupational exposures to specific health consequences. Sources of indoor pollutants are diverse, encompassing human activities, inadequate air exchange, emissions from construction materials, tasks performed indoors (like cleaning or carpeting), and the improper or excessive use of chemical products such as pesticides and disinfectants (Menzies et al., 2003; Park et al., 2004; Syazwan et al., 2012b). Several methodological approaches exist for investigating the interplay between indoor air conditions and sick building syndrome. Among the most prevalent are cross-sectional and cohort studies, which are used to ascertain the relationship between exposure levels and health outcomes, frequently expressed as odds ratios or relative risks (Ahmad & Hassim, 2015). Additional research designs include experimental studies, where specific variables (*e.g.*, ventilation rates) are manipulated to observe their effect on occupant health reports. Case-control studies are typically employed to identify causal factors associated with specific diseases or health manifestations, particularly in the context of epidemics or localized outbreaks (Park et al., 2004). Furthermore, intervention studies, akin to experimental designs but without randomized participant assignment, offer another valuable avenue for investigation. This current research specifically leverages a cross-sectional framework to simultaneously examine environmental exposures and health responses. Effective health protection for building occupants, particularly in educational settings, hinges on vigilant monitoring of environmental and occupational exposure levels. Real-time environmental sensing and data capture of IAQ parameters offer a powerful means for anticipating future problems and conducting retrospective analyses. However, prior IAQ modelling studies and traditional assessment techniques face two critical limitations: high cost and time investment, and reliance on linear models. The substantial time investment and considerable financial outlay associated with renting specialized scientific equipment often pose significant hurdles. These practical constraints can inadvertently contribute to delayed detection and, consequently, more extensive outbreaks or clusters of sick building syndrome. Furthermore, existing IAQ-SBS predictive models often rely on traditional regression techniques, which may struggle with the intricate, non-linear, multi-dimensional relationships inherent in exposure-health data, leading to limited interpretability and lower accuracy. This necessitates the development of an efficient, practical, and highly accurate predictive algorithm for IAQ that moves beyond linear assumptions. Such an algorithmic solution would empower rapid implementation of corrective measures, mitigating the escalation of IAQ-related health ramifications. A promising concept involves creating user-friendly estimation tools that enable non-specialists to initiate prompt action when pollutant concentrations approach predefined thresholds for occupant well-being. Supporting this, some perspectives suggest that structured checklists can serve as essential instruments for comprehensive IAQ management in various indoor environments (Mikut & Reischl, 2011; Syazwan et al., 2012a; Norbäck et al., 2017). The underlying rationale is to safeguard vulnerable populations, such as students, who may exhibit heightened sensitivities to indoor air contaminants.

In higher education settings, students frequently occupy large, often densely populated, learning spaces. Such environments can amplify the susceptibility to health challenges, particularly respiratory conditions and thermal discomfort. These conditions can subsequently impair student concentration, negatively influencing their academic performance, and potentially leading to increased absenteeism or a reduction in motivation due to an unappealing learning atmosphere. Given these realities, there is a pressing scientific need to devise robust methodologies that assist the IAQ sector in comprehending the epidemiology of sick building syndrome and exposure thresholds. This understanding is vital for developing effective predictive tools for health risk assessment, crucial for managing the quality of mechanically supplied indoor air (Norbäck et al., 2017; Syazwan et al., 2013). Therefore, this study aims to develop, evaluate, and ultimately recommend the most precise predictive algorithm, leveraging a multi-model machine learning approach to overcome the limitations of resource-intensive testing and linear modelling, for forecasting the incidence of health complaints, correlating them with specific indoor environmental exposure levels within educational facilities.

## Materials & Methods

This investigation utilized an observational, cross-sectional epidemiological approach within an academic facility located in the Klang Valley, Malaysia. We invited students enrolled at the institute, which offers over 15 distinct courses spanning fields such as health sciences, medicine, epidemiology, and nursing, to participate. Participants were selected *via* random allocation based on their registered courses. The building accommodates approximately 1,500 students, most of whom spend about 7 to 8 h daily indoors for at least three months of their enrollment period (per semester). Prior to the study, building owners, safety and health officers, and building management held a general meeting to brief and advise all potential participants. Comprehensive written and oral information was provided, and informed written consent was obtained from all who chose to take part. Data collection for the study occurred between July 2018 and December 2020, specifically taking place after the peak academic period and before the fasting season.

### Ethics

This study received approval from the Malaysia National Medical Research Registry (NMRR), operating under the Ministry of Health Malaysia (reference number: KKM.NIHSEC. P18-1489(6), dated July 24, 2018; NMRR number: NMRR-17-3449-39116 (Investigator Initiated Research, IIR)). Official authorization to utilize the buildings for this indoor air quality investigation was granted by the management office of the Institut Latihan Kementerian Kesihatan Malaysia (ILKKM) Sungai Buloh (Ministry of Health Malaysia Training Institute of Sungai Buloh). All participants provided valid informed consent by completing a Patient Information Sheet (PIS), in strict adherence to NMRR’s standard operating procedures. Furthermore, explicit permission for the dissemination of findings, including future publications and analysis outputs, was secured directly from the NMRR and subsequently extended to the Director General of Health, Ministry of Health Malaysia.

### Questionnaire and survey form

Participants in this study completed a revised version of the MM040NA questionnaire, a widely recognized instrument for evaluating indoor air quality-related complaints (Syazwan et al., 2012b). This adaptation incorporated elements consistent with the Department of Occupational Safety and Health (DOSH), Malaysia’s Code of Practice on IAQ for Malaysian Use (Department of Occupational Safety Health, 2010). The questionnaire was structured to capture several key areas: sick building syndrome (SBS) symptoms, general health complaints, and perceptions of the indoor environment. Demographic information was also gathered to account for individual variations in subsequent epidemiological analyses. Sick Building Syndrome (SBS) symptoms were quantified using a scoring system: “yes, always (every week)” received two points, “yes, often (every week)” one point, and “no, never” zero points. These individual scores were then totaled, yielding an overall SBS symptom rating ranging from zero to 24. For specific symptom questions (12 in total), a score of two was assigned for “yes, often (every week)”, one for “yes, sometimes (every week)”, and zero for “no, never”. An additional point was added for each of these 12 symptoms if the respondent indicated the symptoms were triggered by an indoor environment. Health complaints (HC) were scored with “Yes” receiving two points and “No” receiving zero. An additional point was added if the issue persisted or occurred within the preceding 12 months. This section covered six specific health issues: asthma, hay fever, allergic reactions affecting the eyes or nose, eczema, respiratory or eye irritation attributed to environmental tobacco smoke (ETS) or strong odors, and common illnesses like colds. The final scores for both SBS and HC were categorized to identify health risk conditions. An SBS score exceeding eight points, or an HC score greater than one point, indicated a health risk. The complete questionnaire, along with the detailed algorithm used for data analysis and modeling in this study, is publicly accessible *via* the GitHub repository at https://doi.org/10.5281/zenodo.17613912.

### Environmental data acquisition and monitoring protocol (IAQ measurement)

The Indoor Air Quality (IAQ) data was systematically gathered, with the sample size determined from estimates in a power calculation. The IAQ data was systematically gathered, with the sample size determined from estimates in a power calculation. Based on an assumed effect size of [0.5], a statistical power of 80%, and a significance level (alpha) of 0.05, the calculation revealed a minimum of 530 participant workspaces were needed for this study. Participants, adhering to established occupational hygiene methodologies (Department of Occupational Safety Health, 2010), were selected. Our sampling strategy involved the random selection of 10 distinct classes from the entire institution, with each class typically accommodating between 30 and 80 students. All selected classes must be equipped with mechanical ventilation air –conditioning system (MVAC) to ensure the study protocol fit with Malaysian standards for comparison purposes. This randomization was achieved through simple random sampling, utilizing computer-generated numbers to select from over 30 available classes within the chosen educational facility. All students within these 10 selected classes were invited to participate, resulting in 513 respondents, representing a 93.3% response rate across these classes. This high response rate minimizes the potential for selection bias among the students in the selected classrooms. All collected respondent data was subsequently randomized into training and testing datasets for the predictive modelling analysis.

The environmental monitoring (exposure assessment) encompassed three primary categories of parameters:

i. Airborne Chemical Contaminants: This included measurements of Formaldehyde (HCHO), Total Volatile Organic Compounds (TVOCs), Carbon Dioxide (CO_2_), and Carbon Monoxide (CO).
ii. Physical Environmental Parameters: These covered ventilation-related elements such as Temperature (Temp), Relative Humidity (RH), Air Movement (Air Mot), and Volumetric Flow Rate (CFM).
iii. Particulate Matter and Count: This involved assessing Particulate Matter 2.5 (PM_2.5_), Particle Count 0.3 (PC_0.3_), Particle Count 0.5 (PC_0.5_), and Particle Count 5.0 (PC_5_).

All data logging occurred at designated points on each respondent’s desk, positioned 110 cm from the floor. Air samples were collected across a minimum of three distinct time intervals throughout the day (morning, noon, and evening). A specialized checklist was employed during the data collection process. Respondent data was gathered purposively, consistent with all obtained permissions and ethical clearances from both participants and the management office. To facilitate spatial analysis, each respondent’s location was systematically mapped using a grid system (*e.g.*, A1, A2, B1, F3) where letters denoted rows and numbers represented columns up to 80). This technique allowed us to understand the spatial variation of indoor measurements, ventilation performance, and health complaints within individual classrooms and across the overall ventilation system.

For ventilation estimation (Cubic Feet per Minute, CFM of air supply from diffusers), measurements were taken at locations nearest to the students, with the data corresponding to the number of students present in the indoor environment. Physical work environment monitoring involved recording air temperature (^o^C), relative humidity (%), and air movement (m/second). Additionally, specific airborne contaminants like carbon dioxide (CO_2_; ppm), carbon monoxide (CO; ppm), total volatile organic compounds (TVOCs; ppm), formaldehyde (ppm), and airborne dust (assessed as particulate matter with an aerodynamic diameter of 2.5- µg/m3 [PM_2.5_- µg/m^3^]) were continuously monitored. Data was logged using an occupational hygiene technique known as surrogate partial period grab samples. Samples were collected over 30-minute intervals at four distinct times of day (morning, afternoon, evening, and late evening) to minimize spatial and temporal variations, thus enhancing data representativeness. Data collection from using similar protocol as describe by Ismail et al. (2022) and Ismail et al. (2025a). For continuous data logging, a specialized suite of occupational hygiene instruments was employed to capture a comprehensive range of environmental factors namely as:

• Gases and thermal parameters: Temperature (^∘^C), Relative Humidity (%), Carbon Dioxide CO_2_, Carbon Monoxide (CO), Formaldehyde (HCHO), and Total Volatile Organic Compounds (TVOCs) were monitored using a single Bacharach IEQ Chek™ 8-Channel IAQ Meter^1^. This meter is equipped with multiple integrated sensors, a data logger, and an internal pump for SD card applications^2^.
• Air velocity and draft: Air movement (m/second) and draft were measured using a TSI Velocicalc Plus 8385 (hot wire anemometer) with LOGDAT1/2 (TSI Inc.), capable of measuring air velocity between 0.01 and 3.00 m/second.
• Particulate matter and count: Airborne dust specifically PM_2.5_ was measured using a Digital Dust Meter (direct reading instrument). Specific particle counts at various diameters (0.3 µm, 0.5 µm, and 5.0 µm) were simultaneously assessed using a KANOMAX Model 3888 Particle Counter.

The traceability of all instruments was maintained, as calibration was strictly performed according to the specified procedures provided by the respective suppliers and manufacturers. For continuous, real-time volatile organic compound (VOC) detection, a specialized Photoionization Detector (PID) sensor was integrated into the Bacharach IEQ Chek™ devices. Precautions were strictly observed when using the real-time photoionization detector, as its readings could be influenced by the presence of non-VOC substances, such as anesthetic or disinfecting gases. Isobutylene (2-methylpropene) served as the reference calibration gas for these real-time monitors (Department of Occupational Safety Health, 2010). Measurements within each classroom were recorded as 5-minute averages throughout the monitoring period, which ran from 8:00 AM to 5:00 PM on each monitoring day. This investigation was meticulously carried out by a Certified Indoor Air Quality Assessor, formally registered with the Department of Occupational Safety and Health, Malaysia (Registration No. JKKP HIE 127/171-4(18)), and its methodology was further validated by a recognized professional IAQ Assessor, Siti Hamimah Ismail.

### Statistical procedure

#### Predictive algorithm, machine learning software and packages

**Figure 1:**
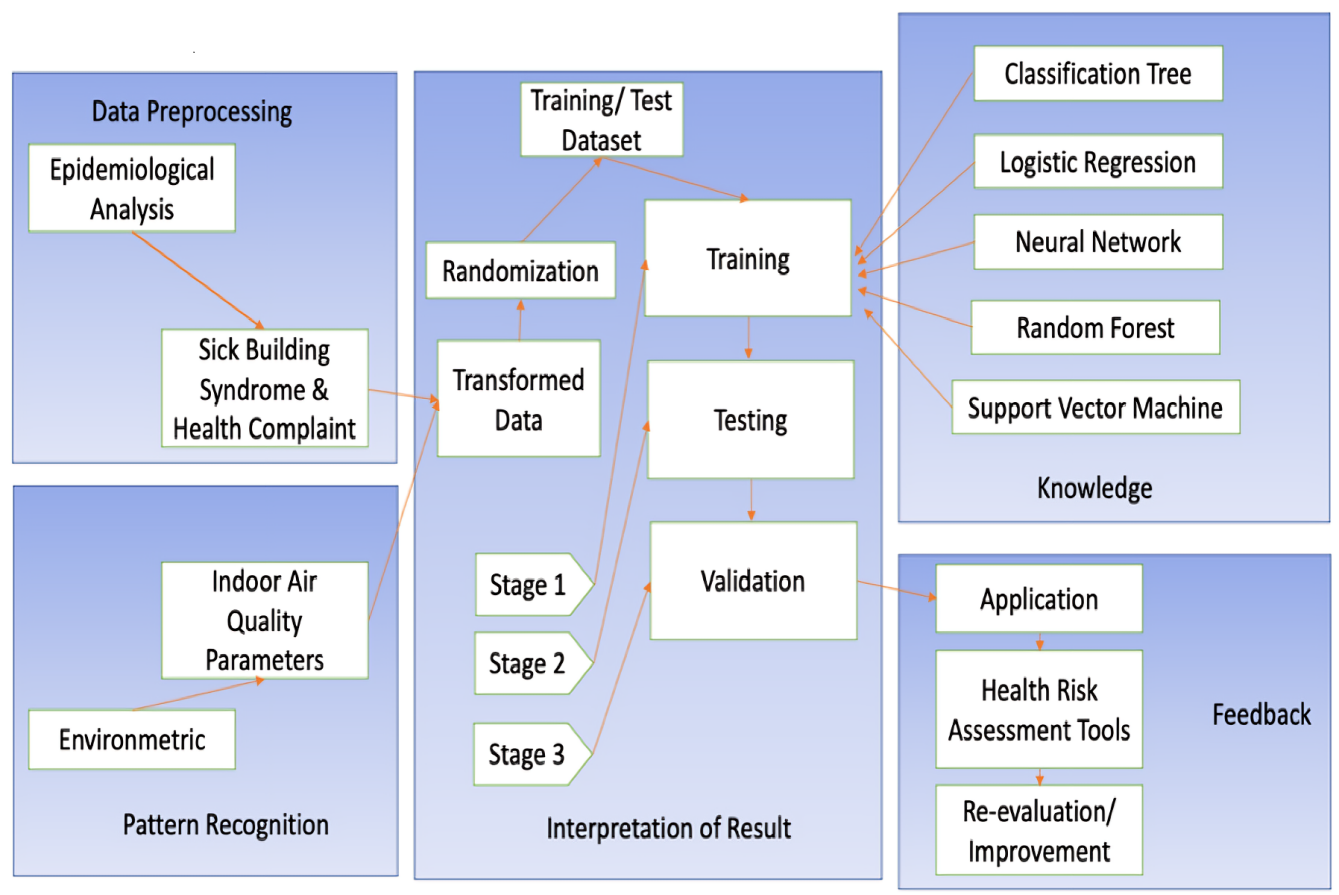
Predictive analytics of health risk assessment tools evaluation for IAQ investigation.

For this investigation, we utilized Orange Data Mining Software Version 3.29.3 (Orange Data Mining, 2013) and IBM SPSS Version 25. Our goal for predictive capability was set at a minimum accuracy level of 70%. Orange Data Mining is an open-source machine learning platform that empowers researchers to construct sophisticated prediction models through its intuitive Graphical User Interface (GUI). It offers a flexible environment for exploring various data analysis methodologies, providing a robust framework for training, testing, and developing predictive models. This software finds extensive application across diverse fields, including genomic research, environmental modeling, biomedicine, quality bioinformatics, and innovation (Orange Data Mining, 2013; McKenna, 2003; Chaves & Marques, 2021). The validation process for our predictive models specifically incorporated IBM SPSS Version 25, leveraging its neural network modeler capabilities. The comprehensive workflow detailing our predictive analytics and validation procedures is illustrated in Fig. 1.

To assess the statistical association between the established exposure classification (High *vs.* Low) and the predicted outcomes for Sick Building Syndrome (SBS) and Health Complaints (HC), the Chi-Square *χ*^2^ test of independence was used. The collected exposure data were categorized into “high” and “low” levels, as detailed in Table 1. This test was applied to the contingency table generated by the correct classification output (Table 2) of each predictive model. The Chi-Square value and corresponding *p*-value were used to demonstrate the statistical significance of the prediction, confirming that the classification accuracy achieved by each algorithm was not due to chance, but represented a genuine association between the exposure score and the predicted health risk category.

**Table 1:** Level of exposure cut-off for the SBS and health.

Parameters (unit)	Cut-off level^*^	Note
HCHO (ppm)	0.013	Based on central tendencies of data and Environmetrics applications
PM_2.5_ (ug/m3)	40	
TVOC (ppm)	0.086	
Air Movement (m/s)	0.091	
CO_2_ (ppm)	780	
Ventilation rate (cfm)	243	
		
SBS Score	More than 8	Based on epidemiological study
Health Score	More than 1	

**Notes.**

* Value was gathered from the Environmetric study (Syazwan et al., 2012a) and epidemiological data analysis (Syazwan et al., 2013).

**Table 2:** Correct classification of the prediction according to the different machine learning algorithm.

ML Algorithm	Correct	%		Misclassify	%	*χ^2^	*p*-value
	Sick building syndrome						
Tree	134	52.34		122	47.66	0.563	0.453
Logistic regression	133	51.95		123	48.05	0.391	0.532
Neural network	124	48.44		128	51.56	0.250	0.617
Random forest	136	53.12		120	46.88	1.000	0.317
SVM	133	51.95		123	48.05	0.391	0.532
	Health complaint						
Tree	178	69.53		78	30.47	39.063	*p* < 0.01
Logistic regression	195	**76.17**		61	23.83	70.141	*p* < 0.01
Neural network	191	74.61		65	23.39	62.016	*p* < 0.01
Random forest	197	**76.95**		59	23.05	74.391	*p* < 0.01
SVM	194	**75.78**		62	24.22	68.063	*p* < 0.01

**Notes.**

ML, Machine Learning (Predictive Algorithm); *Chi, Square testing for the goodness of fit for measuring the distribution of the data across different classification status (correct or misclassify). Bold, high reported classification accuracy in the model.

#### Setup of the predictive analytics approach

Out of the 12 environmental parameters initially recorded in this study, only six were ultimately selected for inclusion in the predictive analytics phase. This targeted approach aligns with findings from prior Environmetric studies, which utilized techniques like Cluster, Discriminant, and Factor analysis to classify environmental exposures (Syazwan et al., 2012a; Syazwan et al., 2013). This classification was determined by analyzing the central tendency of the data, a necessary step given the generally low levels of pollutants observed during these investigations. The six variables identified as “low” or “high” in Table 1 were designated as predictors, while the presence of sick building syndrome (SBS) or health complaints (HC) was set as the target variable. An exposure score, ranging from zero to six, was computed by summing the relevant values. The target variable (dependent variable) itself was binary, categorized as either low risk (“Low”) or high risk (“High”) for SBS or HC. The methodology employed for classifying these predictors mirrors approaches described in several previous research efforts (Tran, Park & Lee, 2020; Xie, Ma & Bai, 2009; Chadha & Mayank, 2016; Tougui, Jilbab & El Mhamdi, 2020).

#### Multiple predictive algorithm models

Our study employed a diverse set of data mining techniques utilizing various predictive classification methods. Specifically, five distinct predictive algorithms were incorporated: Classification Tree (CT), Logistic Regression (LR), Neural Network (NN), Random Forest (RF), and Support Vector Machine (SVM). This comprehensive approach to classification and prediction was critical for mitigating the rigid assumptions often inherent in traditional linear models. The Classification Tree (CT), a member of the supervised learning algorithm family, aims to construct a model capable of forecasting the broad categories of target variables. This algorithm operates by characterizing data records based on their predicted class, initiating a top-down exploration from the root of the tree. It then systematically compares attributes against the record’s characteristics, branching to specific nodes based on these comparisons.

Logistic Regression (LR) was employed for its ability to estimate the likelihood of an event when the target outcome is binary. This method excels at analyzing and predicting outcomes from discrete and ordered data. Another frequently used classification algorithm in predictive analytics is the Neural Network (NN), often referred to as an Artificial Neural Network (ANN). This computational paradigm draws inspiration from the biological neurons found in the brain (Demšar et al., 2013). For the validation phase of our finalized predictive model, we specifically utilized a Multi-Layer Perceptron neural network (Takigawa et al., 2012). The Random Forest (RF) algorithm proved invaluable as it significantly enhances the predictive power of Classification Trees. A key advantage of Random Forest is its inherent robustness against data noise, making it less susceptible to distortions. Finally, the Support Vector Machine (SVM) served as the primary predictive model during the initial exploration and testing phases of this research. This algorithm was particularly crucial for analyzing the intricate, multi-dimensional relationships between our predictors and the target outcome (Takigawa et al., 2012; Mumtaz et al., 2021).

#### Training dataset for analyzing the predictive analytics capacity for exposure and health effects

To prepare the predictive analytics models, the complete dataset from all 513 respondents was randomly divided into training and testing sets using split of 80% training against 20% testing. This random allocation was performed using a computerized number generator. The training dataset comprised 410 instances (80%), while the testing dataset contained 103 (20%). The training data was then used to establish the prediction models within Orange Data Mining, set in prediction mode. The output from the prediction widget was extracted and stored according to the software’s documentation (Orange Data Mining, 2013). Finally, the test dataset was fed into these trained models to evaluate their classification accuracy.

#### Model evaluation for predictive analytics according to classification of accuracy for exposure and health effects

Following the initial prediction modeling in Stage 1 (as depicted in Fig. 1), we assessed the accuracy of the testing dataset. We utilized Orange Data Mining Software’s “Test and Score” widget to determine key performance metrics, including the reliability, precision, and overall accuracy of our predicted models. Both Sick Building Syndrome (SBS) and Health Complaints (HC) models underwent evaluation during this Stage 2 (also shown in Fig. 1). The goal was to identify the most appropriate predictive model for advancement to the subsequent validation phase.

#### Model validation of predictive analytics component between exposure and health effects

The NN algorithm emerged as the most accurate, precise, and reliable predictive model for forecasting health complaints following exposure to specific indoor air quality levels. Consequently, this investigation was extended to a validation phase, utilizing the SPSS Neural Network function in Stage 3 (as shown in Fig. 1). The primary goal of this validation was to confirm the model’s predictive capacity, create graphical representations of its performance, and establish probability values for its application in health risk assessments. Furthermore, the use of SPSS Neural Network allowed us to determine Normalized Importance parameters, which will serve as a scoring method for applying this algorithm in practical scenarios.

### Machine learning model configuration and parameters

To ensure the reproducibility and clarity of the predictive modelling, specific configurations were implemented across the modelling platforms (Orange Data Mining for initial models and IBM SPSS for final validation). The complete dataset was initially partitioned *via* random allocation into an 80% Training set and a 20% Testing set. For the final validated model, the Neural Network (NN) utilized the SPSS Multi-Layer Perceptron (MLP) function with a (6-4-2) architecture, representing six input variables (IAQ exposure factors), four nodes in the hidden layer, and two output categories (Low Risk/High Risk). The Sigmoid activation function was employed in the hidden layer, and training was set to stop after 10 epochs or when a minimal change in error was observed (maximum 1.0E-4). The Random Forest (RF) algorithm was configured using 100 decision trees (estimators), with a limit placed on the maximum depth of the individual trees to prevent overfitting. For the Classification Tree (CT), the Gini index was used for the criterion splitting, and the minimum number of instances allowed in a leaf node was set to 5. The SVM utilized a Radial Basis Function (RBF) kernel due to its effectiveness in analysing the expected non-linear relationships in the environmental health data, with standard regularization and margin parameters applied. Finally, k-fold cross-validation (*k* = 10) was utilized during the model evaluation stage in both Orange Data Mining and SPSS to accurately assess the generalization error and enhance the overall reliability of the models

## Results

### Training of the model

This section delves into the data mining methodologies employed for the observational data gathered during our epidemiological investigation (Syazwan et al., 2012a; Norbäck et al., 2017). We systematically applied five distinct machines learning and predictive analytics tools: CT, LR, NN, RF, and SVM. This comprehensive approach involved training models to evaluate their forecasting capabilities across these various algorithms. The selection of a predictive modeling algorithm often hinges on the specific research strategy adopted by investigators (Ahn et al., 2017; Kourou et al., 2015; Nazir et al., 2015). A particularly robust approach for this planning phase involves classification techniques. Methods such as Cluster, Discriminant, and Factor analysis have proven instrumental in categorizing exposure risk levels within environmental health studies. In this research, we deployed multiple predictive modeling algorithms to pinpoint the most precise predictive capability concerning the incidence of sick building syndrome (SBS) and general health complaints.

**Figure 2:**
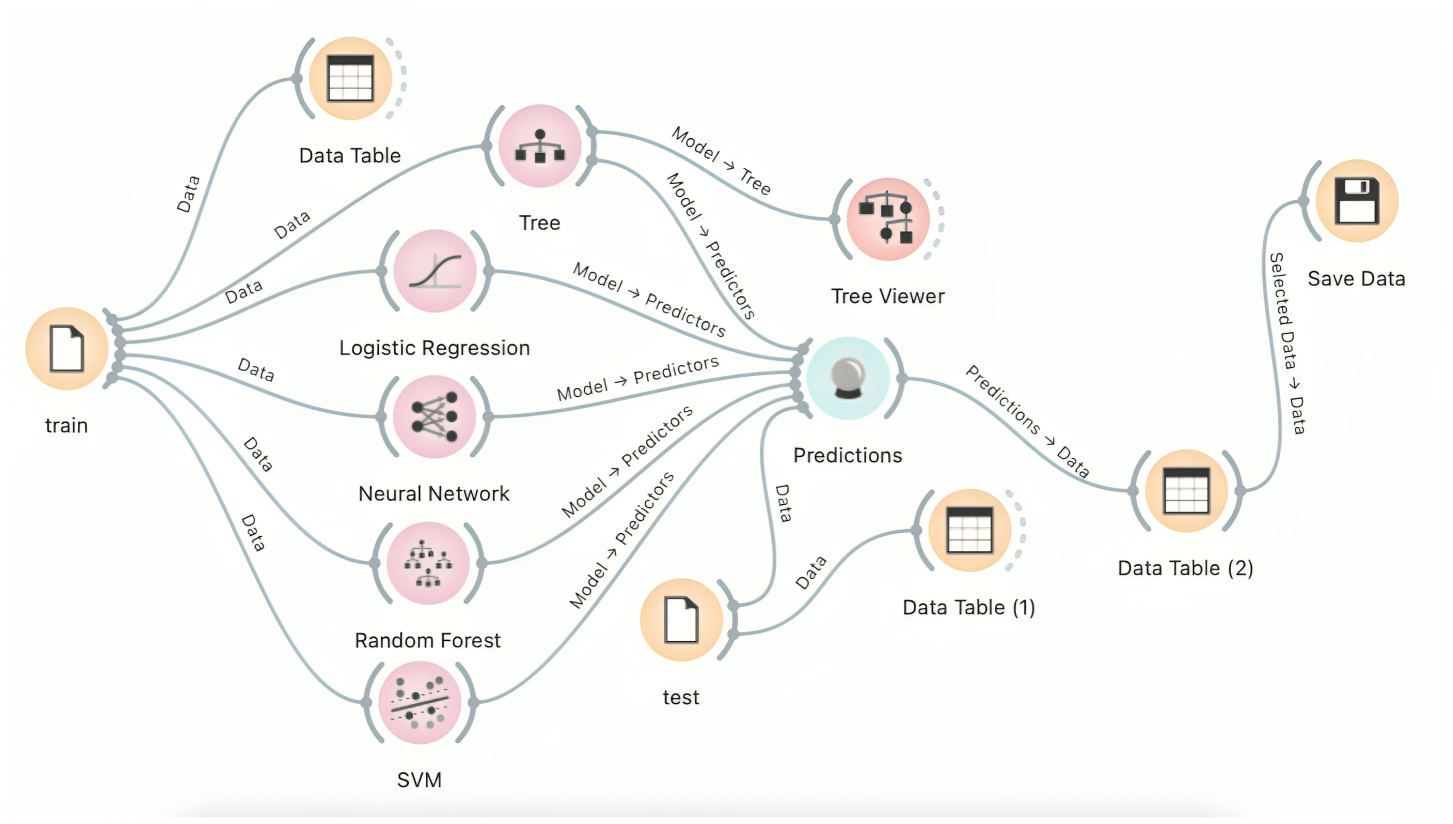
Predictive analytics using multi-model algorithm in predicting the occurrence of Sick Building Syndrome (SBS) and Health Complaint (HC).

Initially, a pre-classification exposure method was used to categorize pollutant levels as either “high” or “low”. This technique, aligning with prior research in the Environmetric field, demonstrated considerable utility (Syazwan et al., 2012a; Ahn et al., 2017; Kourou et al., 2015). By leveraging these pre-classified exposure scores, we then developed a new, integrated score derived from key parameters. This holistic score served as a primary predictor for estimating the risk of SBS and health complaint occurrences. Employing a multi-model predictive algorithm approach allowed us to gain a deeper understanding of underlying patterns and to identify the most accurate algorithm for subsequent detailed evaluation. Figure 2 illustrates the configuration of this multi-modeling function within the graphical software environment. The specific methodology underpinning this approach, which involves similar pattern recognition techniques for detecting health-related events, has been elaborated upon in previous studies (Demšar et al., 2013; Ahn et al., 2017; Kourou et al., 2015; Nazir et al., 2015).

In this investigation, researchers stratified the data analysis by different clusters. This approach directly addressed potential statistical bias within the predicted data. Our results show that the NN algorithm demonstrated the highest correct classification rate for sick building syndrome (SBS) when compared against actual reported data. A similar pattern was observed in the health complaint (HC) data; when analyzed with the exposure score, the NN algorithm again yielded the highest correct classification (see Table 2).

### Evaluation of predictive model in machine learning

**Figure 3:**
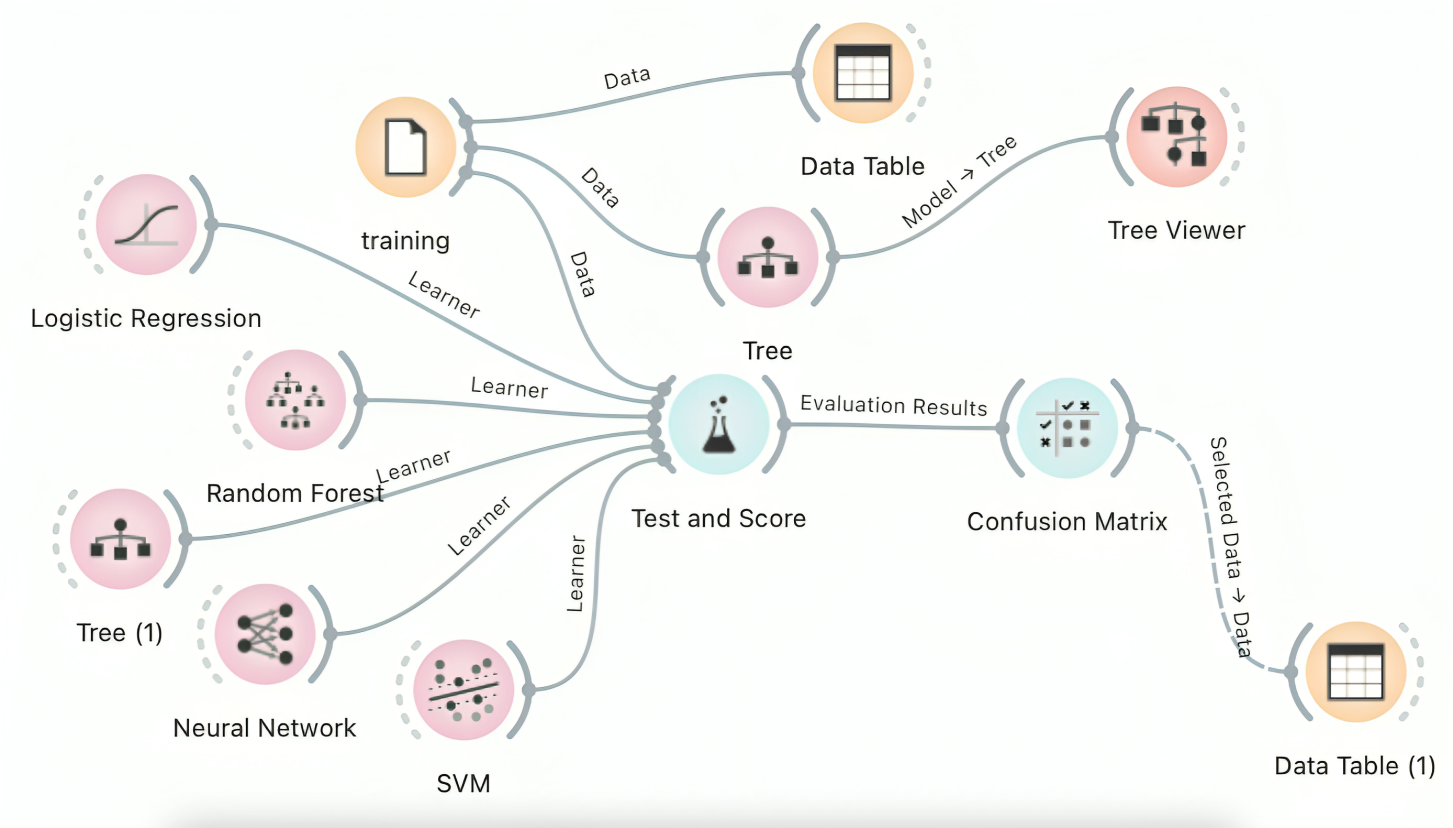
Model evaluation for machine learning of the multi-model algorithm in predicting the SBS and HC.

Once we confirmed the correct classification and compared it across different clusters and condition distributions, we moved on to critically evaluate the model. This involved assessing its classification accuracy and reviewing other objective metrics, including the predictive algorithm indices. We used Orange Data Mining to further analyze the classification accuracy and evaluation indexes reported by the overall algorithm, as shown in Fig. 3.

Our model evaluation results indicate that the predictive capacity and accuracy for SBS are moderate. This suggests the model is suitable for screening purposes or non-critical applications by occupational health professionals and engineers (see Table 3). Comparing different predictive algorithms was crucial to ensuring that our classification accuracy was determined with minimal error or bias (Kourou et al., 2015). While our study reported similar indices to previous experiments (Nazir et al., 2015; Li et al., 2012; Challoner, Pilla & Gill, 2015), the specific values weren’t as high. We attribute this difference to our use of an observational study method in a real-world setting, as opposed to a controlled laboratory experiment. This finding is consistent with observations from studies conducted in commercial buildings (Xie, Ma & Bai, 2009).

**Table 3:** Comparison of the accuracy indexes for classification of the different machine learning algorithm for SBS and HC.

ML model^*^	**AUC**	**CA**	**F1**	**Precision**	**Recall**
Sick Building Syndrome (SBS)					
Logistic regression	0.508	0.499	0.499	0.498	0.499
Tree	0.517	0.509	0.508	0.516	0.509
SVM	0.511	0.501	0.501	0.501	0.501
Neural network^ˆ^	0.554	0.536	0.537	0.539	0.536
Random forest	0.571	0.536	0.537	0.540	0.536
Health Complaint (HC)					
Logistic regression	0.559	0.728	0.632	0.681	0.728
Tree	0.549	0.647	0.642	0.637	0.647
SVM	0.589	0.711	0.649	0.647	0.711
Neural network^ˆ^	0.548	0.726	0.660	0.676	0.726
Random forest	0.605	0.711	0.670	0.664	0.711

**Notes.**

ML, Machine Learning (Predictive Model).

* Model was arranging in highest sequence order from the Classification Accuracy.

ˆSelected model based on the criteria for highest predictive capacity.

### Model validation of selected predictive analytics algorithm

Based on the research findings, we conducted further analysis using statistical software to precisely determine the finalized predictive capacity and accuracy index. This step was essential to ensure our methodology could be effectively applied to manage indoor air quality issues in real-world settings. After performing model prediction on both training and testing datasets, and then evaluating the model on the testing data, our validation process specifically focused on health complaints (HC). This predictive component demonstrated consistently high accuracy in forecasting health events, more so than SBS. Consequently, a Multi-Layer Perceptron NN algorithm within SPSS was chosen for validation. This allowed us to ascertain the percentage of correct classifications for the previously tested data. For this validation exercise, the SPSS Neural Network function utilized a 80% training set and a 20% testing set (Liu et al., 2018; Kim et al., 2018).

Once we confirmed the predictive algorithm’s superior classification and accuracy, the model was integrated into the validated framework within the SPSS Neural Network algorithm. The final neural network configuration, represented by its nodes, was (6-4-2), corresponding to six input variables, four hidden layers, and two output categories. As presented in Table 4, the percentage of correct classifications exceeded 70%, which is considered acceptable (Saad et al., 2015; Lei et al., 2019). This finding is consistent with a prior experimental study that validated a Neural Network algorithm for assessing indoor pollutants using electronic sensors (Nazir et al., 2015). Our emphasis on health complaints for predictive evaluation underscores its significance in analyzing exposure-health relationships.

**Table 4:** Percentage of correct classification of the Neural Network model in predicting the outcome (SBS and HC) from the exposure score (high and low indoor air quality levels).

Model validation			Healthy	Risk	% CC^ˆ^
For SBS	Testing period	Training	0	217	47.1
		Testing	0	56	
	Validation	Training	0	193	45.6
		Testing	0	47	
For HC	Testing period	Training	298	0	72.7
		Testing	76	0	
	Validation	Training	112	0	73.8
		Testing	27	0	

**Notes.**

ˆCC = Percentage of Correct Classification (Overall).

### Practical application of the neural network for indoor air quality diagnosis for health complaint prediction

Leveraging neural networks as a predictive tool for diagnosing health risks is incredibly valuable for technical professionals in the field. This algorithmic approach empowers engineers, industrial hygienists, and occupational health management teams to effectively identify and address issues related to indoor air quality and ventilation within workspaces.

To boost the practical application of this neural network algorithm, we used a classification and scoring technique. This allowed us to explore how different levels of measured indoor air quality correlate with potential health outcomes or effects. By quantifying the percentage and probability of health outcomes, investigators can now gain clearer insights into health risk conditions in suspected or problematic areas. The probability scores and their distribution across various exposure levels are detailed in Table 5. This method offers a significant advantage over previous studies that primarily focused only on measured chemical levels (Saad et al., 2015; Lei et al., 2019; Khazaei, Shiehbeigi & Kani, 2019).

**Table 5:** Estimated exposure and predictive health risk element for indoor air quality investigation applications.

Exposure score^*^	Health status	Frequency	Percent (%)	Mean probability	SD probability	^ˆ^Risk cat
0	Healthy	4	100	0.6732	0.02836	1
1	Healthy	49	98	0.7588	0.11108	
	Health risk	1	2			
2	Healthy	33	71.7	0.6802	0.20895	2
	Health risk	13	**28.3**			
3	Healthy	71	80.7	0.6944	0.19276	
	Health risk	17	**19.3**			
4	Healthy	25	71.4	0.6203	0.32335	
	Health risk	10	**28.6**			
5	Healthy	16	100	0.7559	0.03421	3
6	Healthy	17	100	0.6667	0	

**Notes.**

* Exposure score was rated as 1 if determined higher than the cut-off; The focus of the exposure score that is possible to be used as indicator will be exposure score of 2, 3 and 4 (bold).

ˆRisk Cat = Risk category (This element can be a definite tools for investigator for justifying the overall assessment performed during investigation): 1 = The health risk for this areas was assume to be low and the health effects possibly due to non-indoor air quality factors (excluding ergonomics issues); 2 = health risk for this areas was considered significant to cause the health issues (further investigation needed on controlling and managing exposure, personnel survey was optional); 3 = health risk for this areas was consider unknown/ non-conclusive, detail occupational health assessment needed (personnel survey is required).

**Figure 4:**
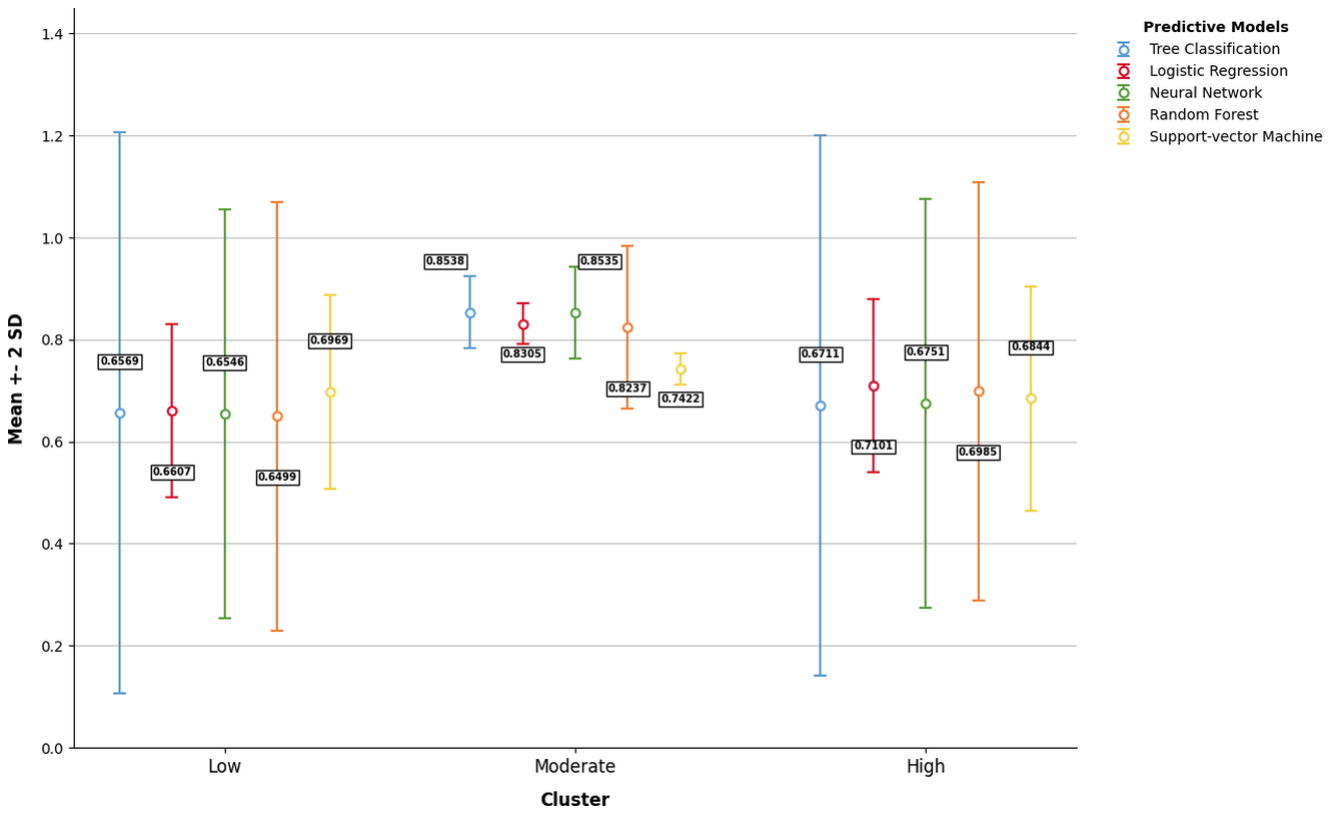
Mean distribution of the classification accuracy index for testing/ validation according different predictive model.

**Figure 5:**
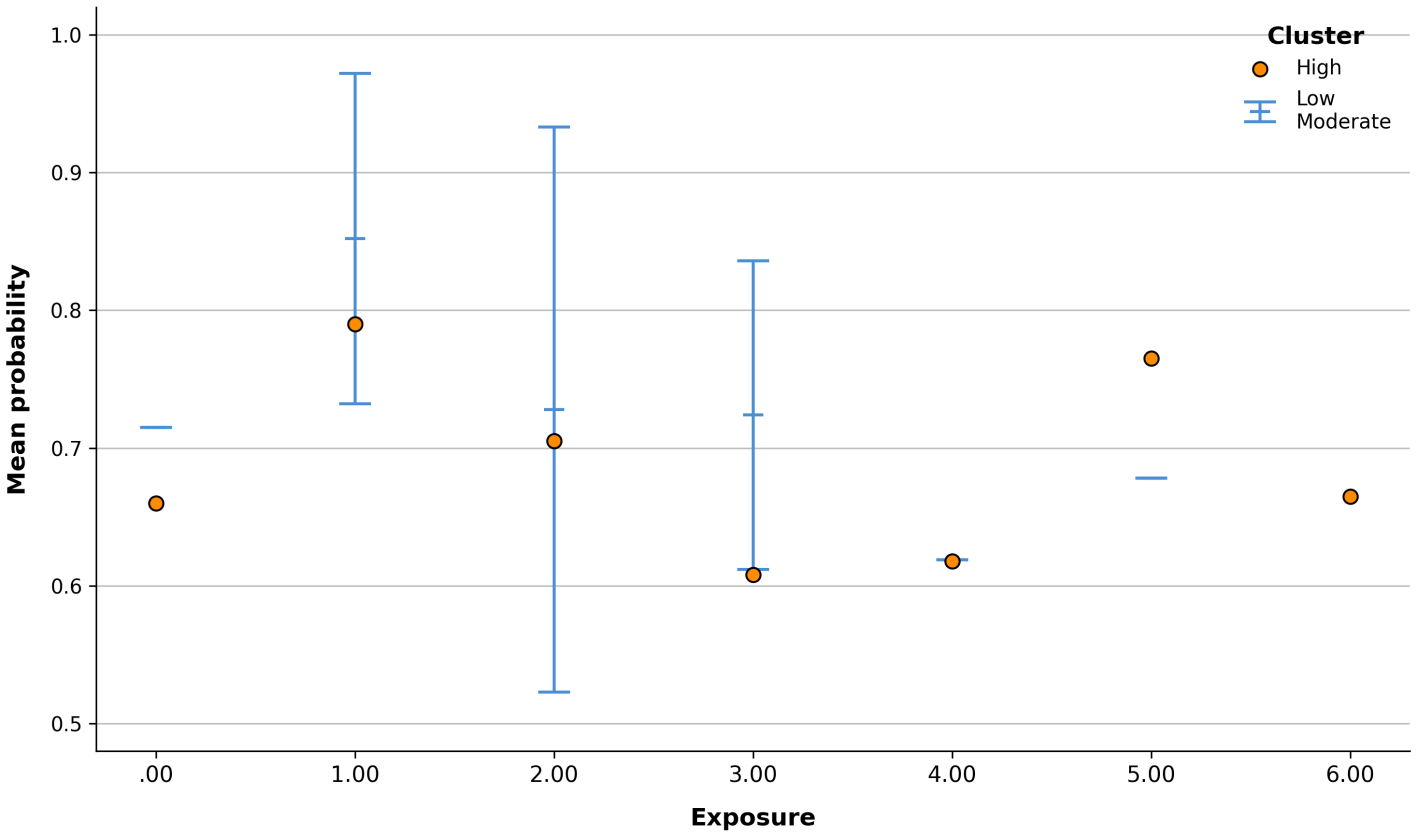
High Low probability with exposure status among different cluster.

Based on the data presented in Table 4, Figs. 4 and 5, we’ve established the mean probability of health complaint occurrences. This allows investigators to quickly grasp the severity of issues and prioritize effective indoor air quality management strategies. The data in Fig. 4 is segregated by cluster, further refining the exposure classification technique previously explored in another study (Syazwan et al., 2013). By understanding the probable health outcomes within primary clusters through the exposure score, investigators can spatially pinpoint problem areas, enabling prompt resolution.

### Practical application for predictive algorithm to be implemented in IAQ investigation

**Figure 6:**
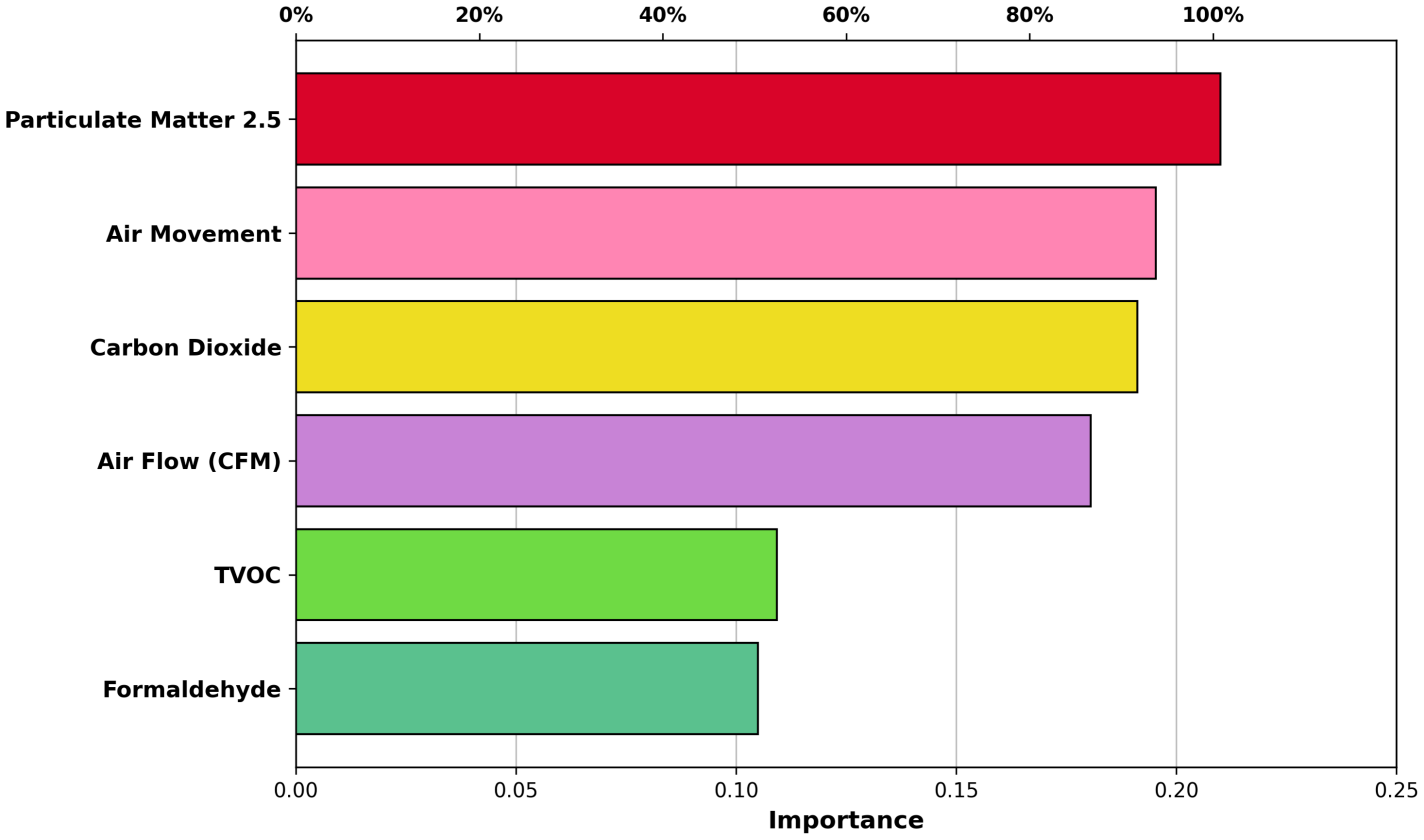
Normalized importance for the predicted parameters in neural network predictive algorithm.

The findings from this investigation offer a robust framework for classifying and predicting potential health outcomes based on the exposure levels identified during initial indoor air quality screenings. To ensure this predictive model could function as a reliable algorithm, we estimated the normalized importance of independent variables using SPSS Multi-Layer Perceptron. The results, displayed in Fig. 6, indicate the most influential parameters, listed here in ascending order of importance: PM_2.5_, Air Movement, Carbon Dioxide, Ventilation Rate, TVOC, and Formaldehyde. This hierarchy highlights which factors are most critical in determining health effects. Furthermore, the simulated data for use with the algorithm is provided in Table 6.

**Table 6:** Simulated data collection and their prediction of health status.

Exposure	Recorded data	Classification	Score	Interpretation^*^
HCHO	0.1	High	1	This area was able to report at least 19% of health complaints from overall occupants. *E.g.,* if the areas were occupied by 10 persons, 2 of them were estimated to have significant health issues during their stay indoors.
PM_2.5_	20	Low	0	
TVOC	0.1	High	1	
Air Movement	0.1	Low	0	
CO_2_	800	High	1	
Airflow	300	Low	0	
		Total	3	

**Notes.**

* Interpretation was made based on mean probability on Fig. 4 and predictive health risk on Table 5.

## Discussion

This study pioneers the application of machine learning algorithms in classifying indoor air quality (IAQ) risk, specifically focusing on the occurrence of sick building syndrome (SBS) among occupants. Building upon prior environmetrics research that introduced the use of carbon dioxide as a surrogate measure for IAQ classification based on checklists (Syazwan et al., 2012b; Mikut & Reischl, 2011; Syazwan et al., 2012a), our investigation similarly mapped exposure assessment data from IAQ monitoring to collective symptomatic scores. This approach successfully differentiated potential problematic IAQ conditions using machine learning. By leveraging simulated data and predicting health status, occupational health professionals can propose immediate interventions if the exposure score reaches or exceeds 3.

For educational facilities and workspaces, where occupants spend significant time indoors, a tool capable of predicting potential health issues in specific indoor areas is essential. A delayed response or prolonged validation of exposure-outcome analyses from detailed IAQ assessments can lead to severe health consequences if immediate vigilance and control measures are not implemented (Syazwan et al., 2012a; Norbäck et al., 2017; Syazwan et al., 2013). Therefore, when applying data mining techniques to predict IAQ conditions, the highest possible accuracy is paramount. This necessitates selecting the correct predictive algorithm to ensure precise discrimination of health issues, thereby avoiding misclassification. A scenario where false negatives outweigh false positives in the testing group (Tougui, Jilbab & El Mhamdi, 2020; Demšar et al., 2013) could lead to inaccurate IAQ diagnoses, posing significant health risks to current or future occupants.

The use of an epidemiological approach to diagnose exposure levels linked to health complaints is a novel contribution to the fields of occupational health and mechanical engineering. While numerous medical studies have successfully employed machine learning and data mining to predict disease or health outcomes, including in diabetic studies (Tougui, Jilbab & El Mhamdi, 2020; Gan et al., 2021), our proposed neural network predictive algorithm demonstrated acceptable performance when compared with previous IAQ status studies that relied on regression or logistic regression models (Park et al., 2004; Norbäck et al., 2017). Our study achieved a correct classification rate exceeding nearly 75% using neural networks. Subsequent analysis of these results involved a discriminative approach to further classify health issue status based on specific percentages of correct classification. Although our classification accuracy was somewhat lower than some previous studies (Kourou et al., 2015; Zhang et al., 2014), the strength of our investigation lies in its direct linkage to epidemiological data collected during the exposure assessment period (Syazwan et al., 2013).

Furthermore, our study meticulously controlled for statistical and selection bias by randomly allocating overall data into separate training, testing, and validation datasets. This rigorous approach, where the entire dataset was randomly partitioned, effectively mitigated potential biases, a strategy supported by previous research advocating for varied data components to prevent selection bias in final models (Lei et al., 2019; Zhang et al., 2014). The concept of pre-classified levels introduced in this study can be adapted for comparative analysis across different population groups. This will empower engineers and hygienists to make informed inferences based on exposure levels recorded in their respective premises. To ensure the robustness of our predictive model and to guard against overfitting and underfitting, we incorporated k-fold Cross-validation during both the model evaluation phase in Orange Data Mining Software and the SPSS Neural Network data analysis stage. This method provides confidence that the statistical biases associated with neural network algorithms can be more accurately interpreted, leading to reduced error in the output. Such strategies have been recently employed in modeling studies of carbon dioxide parameters (Gan et al., 2021), where carbon dioxide serves as an indicator for assessing ventilation efficiency. A high reading from an infrared sensor, for example, can signal slower or inefficient mechanical ventilation, prompting further investigation by IAQ assessors.

A critical component of our multi-model strategy was the comparison of quantitative performance metrics (Table 3). When evaluating the models for the primary outcome, HC, RF, and NN algorithms demonstrated superior overall performance metrics. Specifically, the RF model achieved the highest Area Under the Curve (AUC) at 0.605 and the highest F1-Score at 0.670, indicating the best balance between precision and recall, suggesting strong reliability. The NN model followed closely with an AUC of 0.548 and an F1-Score of 0.660, maintaining the second-highest Classification Accuracy (CA) at 0.726. The lower performing models, such as Logistic Regression and Tree, exhibited lower AUCs and F1-Scores, confirming their struggles with the complex, non-linear epidemiological data. The superior performance of the RF and NN algorithms validates our use of advanced machine learning to overcome the limitations of traditional linear models, which are often inappropriate for this multi-dimensional, real-world data. For practical application, the high Recall and Precision scores of the NN and RF models are vital: high Precision ensures minimal false alarms for IAQ engineers, while high Recall (Sensitivity) ensures that actual health risks are rarely missed, positioning these models as highly dependable diagnostic tools for immediate intervention.

Through a systematic evaluation of existing literature and the results from our experiments, we affirm that the Neural Network is the most competent classification technique for predicting indoor air quality exposure outcomes. However, it’s crucial to acknowledge that while this method proved optimal for this specific research, different datasets or exposure levels may necessitate adjustments or alternative algorithms. To foster collaborative research, we are making our model accessible *via* an open-access repository. This repository includes syntax that allows researchers from other disciplines to modify the established levels or algorithms to enhance the predictive capacity of SBS and HC in occupational health and mechanical engineering applications.

The substantial size and suitability of our dataset, with a proportional 50% allocated for training and testing, aligns with epidemiological approaches for case-control study designs. This partitioning was vital to avoid artificially inflating odds ratios, which can occur if logistic regression is used for model training and evaluation (Saad et al., 2015; Saini, Dutta & Marques, 2020). With the current trend of continuous data collection by assessors, the algorithm developed in this study can be further integrated with real-time IAQ monitoring devices for ongoing health issue prediction. While our hygienic approach focused on regulatory requirements and the ICOP-IAQ standard, making the established levels applicable primarily for 8-hour operational interpretations (Department of Occupational Safety Health, 2010), future studies could extend this algorithm for a holistic assessment of air conditioning performance in conjunction with IAQ levels by leveraging continuous monitoring devices connected to the Internet of Things (Ahn et al., 2017).

Despite potential limitations from restricted access to open-source IAQ monitoring data and professional profiles from regulators, our predictive modeling offers significant value. Using educational buildings, with students as key occupants, provides an important benchmark for IAQ engineers and hygienists, offering insights applicable to large, open-plan workspaces. Although Malaysia’s current Personal Data Act (PDA) might pose future challenges for the broader implementation of such predictive models, the concept of a collective repository for IAQ data remains essential for assisting building owners in managing evolving IAQ risks. In the context of the COVID-19 pandemic, this study aids engineers and building managers in identifying potentially unhealthy areas, enabling proactive mitigation to prevent outbreaks. We believe that sharing this predictive algorithm can revolutionize environmental and occupational health by integrating an engineering mindset. Furthermore, mechanical engineers can benefit by scheduling predictive maintenance to pre-empt health issues, and both engineers and hygienists can leverage this research for forensic investigations during outbreaks in offices or other indoor environments, as reported by previous researchers (Enzevaee, Mailah & Kazi, 2018; Sulaiman et al., 2011; Nur Zakri & Ahmad, 2019).

The exposure levels established in this study are considered low and highly sensitive, yet they powerfully demonstrate that pollutants generated by indoor activities significantly impact occupant health. The integration of epidemiological, engineering, and quality analysis in our results will support future research endeavors in this domain. Machine learning is increasingly vital for building diagnostics, especially in highly occupied structures. In this era of modern building construction and widespread use of mechanical ventilation as the primary “breathing” mechanism for buildings, this algorithm can be further integrated with Building Information Modeling (BIM) to efficiently monitor supplied air (Zhang et al., 2014; Bakht et al., 2022; Latoń et al., 2025; Fan & Ding, 2022). The complexities of investigating SBS and health complaints due to occupational exposure in indoor environments can be simplified using predictive algorithms that can identify the number of occupants requiring administrative control to prevent occupational health litigation. This study suggests that the neural network machine learning algorithm can serve as an invaluable tool for IAQ investigators, facilitating conclusive and timely health risk assessment processes (Bakht et al., 2022; Norbäck et al., 2017; Ismail et al., 2025b; Ismail & Amiruddin, 2025). However, it is imperative that this algorithm does not replace the need for occupational health professionals to conduct air sampling or IAQ audits, as their expert interpretation is crucial for delivering definitive findings to building owners. The exploration of Orange Data Mining software signifies a new and important element in machine learning and predictive algorithms, with numerous open-source data mining software options available for predictive analytics (Orange Data Mining, 2013; Chaves & Marques, 2021; Xie, Ma & Bai, 2009; Garcia-Pinilla, Jurio & Paternain, 2025; Mazoteras-Pardo et al., 2023; Neo et al., 2023). Consequently, this study explores the potential for engineers to incorporate predictive analytics into the operational management of buildings equipped with mechanical ventilation. The drag-and-drop graphical flow coding technique simplifies manipulation and updates to monitoring levels for users and future researchers. Our open-source repository encourages future researchers to update and share algorithm adjustments during the training phase. This collaborative effort is intended to unite epidemiologists and engineers in preventing health issues arising from modern building usage. Amidst the COVID-19 pandemic (Garcia-Pinilla, Jurio & Paternain, 2025), this algorithm aims for broader application in commercial buildings, ultimately helping to reduce health complaints and SBS symptoms to acceptable levels.

The findings from this investigation offer a robust framework for classifying and predicting potential health outcomes based on the exposure levels identified during initial indoor air quality screenings. To ensure the model’s interpretability and practical utility, we estimated the variable importance ranking using the Normalized Importance parameter from the SPSS Multi-Layer Perceptron. The results, displayed in Fig. 6, indicate the most influential parameters, listed here in ascending order of importance: PM_2.5_, Air Movement, Carbon Dioxide (Garcia-Pinilla, Jurio & Paternain, 2025; Ismail et al., 2025b), Ventilation Rate, Total Volatile Organic Compounds (TVOC), and Formaldehyde. Practical Implications for IAQ Engineers and Hygienists: This precise variable ranking moves the model beyond being merely diagnostic by directly informing engineering intervention. IAQ professionals can utilize this hierarchy to prioritize corrective measures (*e.g.*, focusing mitigation efforts immediately on reducing Formaldehyde and TVOC, which are the highest predictors) rather than dedicating resources equally across all parameters. Furthermore, the model’s spatial data linkage allows engineers to quickly pinpoint and isolate problem zones utilizing dashboard or environmental health digital approach (Ismail & Amiruddin, 2025), streamlining predictive maintenance schedules and ensuring prompt resolution where the health risk is highest.

### Methodological consideration

This study took place in an educational setting, employing an observational design within classrooms equipped with mechanical ventilation systems. These systems were managed and controlled by external contractors, which meant some inherent bias or limitations in fully controlling the climate could exist. We mitigated this by performing repetitive indoor air quality measurements in the study areas at least three times a day, over the course of a week. This ensured the validity of our findings across both spatial and temporal variations.

We assessed SBS using a validated questionnaire. While the potential for misreporting or under-reporting of symptoms always exists, we addressed this by incorporating objective health evaluations using the Sick Building Utility Test (SBUT) and diagnosing related health complaints. This dual approach allowed SBS and health outcomes to serve as reliable health indicators for our predictive analytics.

A major strength of this work is its comprehensive monitoring of multiple environmental factors, including gases, chemicals, and particulates. This robust data collection helped verify ventilation performance and identify pollutant pathways, offering a representative view of key problems in a mechanically ventilated educational building. Another significant strength was the high participant response rate, which greatly reduced the likelihood of selection bias. Conducted in a higher education setting, this study of a building serving many occupants over time can serve as a reliable standard for monitoring and setting action levels. Our study provides valuable information on indoor air quality levels, associated health impacts, machine learning algorithms, classification accuracy, and the probability of risk influencing health effects within an educational facility.

While the 73.8% accuracy is acceptable for a preliminary screening tool, it acknowledges a misclassification rate of approximately 26%. In a real-world health context, this necessitates a balanced consideration of false positives and false negatives to avoid potential alarm fatigue or missed diagnoses, underscoring the need for continuous model improvement and retraining with larger datasets. Beyond its strategic use of personal exposure measurements, which significantly enhanced prediction accuracy, the study’s commitment to openly sharing its algorithm and analytics design further bolsters its strength. The algorithm is readily available for future researchers to adjust, refine, cross-validate, and apply with other parameters, such as microbial contaminants and thermal comfort.

## Conclusions

This study confirms the robust potential of data mining techniques, specifically machine learning algorithms, for predictive diagnostics in environmental health and building engineering. The NN algorithm was identified as the most effective classifier, providing a preliminary and objective diagnosis of occupant health complaints based on quantified exposure scores from field monitoring. The model accurately predicted at least 298 out of 410 instances, achieving a classification accuracy of 73.8%. This performance, rigorously verified using k-fold cross-validation, validates the necessity of utilizing advanced non-linear modelling techniques for complex epidemiological data. The primary contribution of this research is the establishment of a quantifiable, reliable, and scalable predictive framework. This tool offers building management and occupational health professionals the capacity for early detection of health risks, enabling proactive intervention and mitigating severe health consequences associated with mechanically ventilated environments. Future research is directed toward applying and refining this predictive algorithm in real-world operational scenarios. This next step requires the integration of the model with continuous monitoring and Internet of Things (IoT) technology to facilitate live risk predictions and automated warnings, moving building diagnostics from retrospective assessment toward dynamic, preventative health management.

## Supplemental Information

10.7717/peerj.20964/supp-1 Supplemental Information 1 STROBE Checklist (Cross Sectional Study)

## Additional Information and Declarations

### Data Availability

The following information was supplied regarding data availability: The complete questionnaire, along with the detailed algorithm used for data analysis and modeling in this study, is available at GitHub and Zenodo: - https://github.com/ismailsakdo/iaqkkm/tree/v1.0.0-iaq-sbs-drsai. - Kajidataonline. (2025). ismailsakdo/iaqkkm: Official Release: V1.0.0 - Predictive NN Model for IAQ & Health Complaints (v1.0.0-iaq-sbs-drsai). Zenodo. https://doi.org/10.5281/zenodo.17613912.
