# Supplementary material for: Application of machine learning algorithms in building health diagnostics: predictive analytics evaluating indoor air quality and sick building syndrome in educational settings
Source: PeerJ. 2026 Jul 9;14:e20964. doi: 10.7717/peerj.20964 (PMC13356826; doi:10.7717/peerj.20964)
Supplement: Supplemental Information 1 [file peerj-14-20964-s001.docx]

STROBE Statement—checklist of items that should be included in reports of observational studies

|  | Item No. | Recommendation | Page  No. | Relevant text from manuscript |
| --- | --- | --- | --- | --- |
| **Title and abstract** | 1 | (*a*) Indicate the study’s design with a commonly used term in the title or the abstract | 2 | We achieved this by gathering and analyzing epidemiological and exposure assessment data via cross-sectional study approach |
|  |  | (*b*) Provide in the abstract an informative and balanced summary of what was done and what was found | 2 | Same as above |
| Introduction | | | |  |
| Background/rationale | 2 | Explain the scientific background and rationale for the investigation being reported | 4 | Among the most prevalent are cross-sectional and cohort studies, which are used to ascertain the relationship between exposure levels and health outcomes, frequently expressed as odds ratios or relative risks |
| Objectives | 3 | State specific objectives, including any prespecified hypotheses | 5 | Therefore, this study aims to develop, evaluate, and ultimately recommend the most precise predictive algorithm for forecasting the incidence of health complaints, correlating them with specific indoor environmental exposure levels within educational facilities. |
| Methods | | | |  |
| Study design | 4 | Present key elements of study design early in the paper | 5 | This investigation utilized an observational, cross-sectional epidemiological approach within an academic facility located in the Klang Valley, Malaysia. |
| Setting | 5 | Describe the setting, locations, and relevant dates, including periods of recruitment, exposure, follow-up, and data collection | 5 | This investigation utilized an observational, cross-sectional epidemiological approach within an academic facility located in the Klang Valley, Malaysia. We invited students enrolled at the institute, which offers over 15 distinct courses spanning fields such as health sciences, medicine, epidemiology, and nursing, to participate. |
| Participants | 6 | (*a*) *Cohort study*—Give the eligibility criteria, and the sources and methods of selection of participants. Describe methods of follow-up  *Case-control study*—Give the eligibility criteria, and the sources and methods of case ascertainment and control selection. Give the rationale for the choice of cases and controls  *Cross-sectional study*—Give the eligibility criteria, and the sources and methods of selection of participants | 5 | Participants were selected via random allocation based on their registered courses. The building accommodates approximately 1500 students, most of whom spend about 7 to 8 hours daily indoors for at least three months of their enrollment period (per semester). |
|  |  | (*b*) *Cohort study*—For matched studies, give matching criteria and number of exposed and unexposed  *Case-control study*—For matched studies, give matching criteria and the number of controls per case |  |  |
| Variables | 7 | Clearly define all outcomes, exposures, predictors, potential confounders, and effect modifiers. Give diagnostic criteria, if applicable | 6  5 | The environmental monitoring (exposure assessment) encompassed three primary categories of parameters;  This adaptation incorporates components that are relative to the Department of Occupational Safety and Health (DOSH), the Code of Practice on indoor air quality in Malaysia used by Malaysians |
| Data sources/ measurement | 8* | For each variable of interest, give sources of data and details of methods of assessment (measurement). Describe comparability of assessment methods if there is more than one group | 6 | The complete questionnaire, along with the detailed algorithm used for data analysis and modeling in this study, is publicly accessible via the GitHub Repository at https://github.com/ismailsakdo/iaq. |
| Bias | 9 | Describe any efforts to address potential sources of bias | 16 | The identification of Sick Building Syndrome (SBS) was done using a validated peer-reviewed questionnaire, accompanied by a compound of objective health measurements. The use of self-reports is not free of recall bias but they were supplemented by Sick Building Utility Test (SBUT) which is an in-situ diagnostic that helps to correlate the self-reported complaints associated with SBS with physiological indices. |
| Study size | 10 | Explain how the study size was arrived at |  |  |

Continued on next page

| Quantitative variables | 11 | Explain how quantitative variables were handled in the analyses. If applicable, describe which groupings were chosen and why | 6 | Procedural sampling involved approaching 10 unique classrooms based on a statistical sampling design whereby simple random sample was used as a result of computer- generated numbers accessing more than 30 available rooms in the chosen learning institution. All students within these 10 selected classes were invited to participate, resulting in a 100% response rate from all respondents across these classes. All collected respondent data was subsequently randomized into training and testing datasets for the predictive modeling analysis. |
| --- | --- | --- | --- | --- |
| Statistical methods | 12 | (*a*) Describe all statistical methods, including those used to control for confounding | 8 | The research study used Orange Data Mining Software Version 3.29.3 and IBM SPSS Version 25, which acted as its reference benchmark to compare the other. Predictive capability was benchmarked to minimum accuracy of 70 %; the results produced by the two systems surpassed this requirement. |
|  |  | (*b*) Describe any methods used to examine subgroups and interactions | 8 | The validation process for our predictive models specifically incorporated IBM SPSS Version 25, leveraging its neural network modeler capabilities. The comprehensive workflow detailing our predictive analytics and validation procedures is illustrated in Figure 1. |
|  |  | (*c*) Explain how missing data were addressed | n/a | Response rate is 100% |
|  |  | (*d*) *Cohort study*—If applicable, explain how loss to follow-up was addressed  *Case-control study*—If applicable, explain how matching of cases and controls was addressed  *Cross-sectional study*—If applicable, describe analytical methods taking account of sampling strategy | 6 | Procedural sampling involved approaching 10 unique classrooms based on a statistical sampling design whereby simple random sample was used as a result of computer- generated numbers accessing more than 30 available rooms in the chosen learning institution. All students within these 10 selected classes were invited to participate, resulting in a 100% response rate from all respondents across these classes. All collected respondent data was subsequently randomized into training and testing datasets for the predictive modeling analysis. |
|  |  | (*e*) Describe any sensitivity analyses | n/a |  |
| Results | | | | |
| Participants | 13* | (a) Report numbers of individuals at each stage of study—eg numbers potentially eligible, examined for eligibility, confirmed eligible, included in the study, completing follow-up, and analysed | 6 | All students within these 10 selected classes were invited to participate, resulting in a 100% response rate from all respondents across these classes. All collected respondent data was subsequently randomized into training and testing datasets for the predictive modeling analysis. |
|  |  | (b) Give reasons for non-participation at each stage | n/a |  |
|  |  | (c) Consider use of a flow diagram | n/a |  |
| Descriptive data | 14* | (a) Give characteristics of study participants (eg demographic, clinical, social) and information on exposures and potential confounders | 8&9 | Table 1 and Table 2 (Overall SBS) |
|  |  | (b) Indicate number of participants with missing data for each variable of interest | 8&9 | Table 1 and Table 2 (Overall SBS) |
|  |  | (c) *Cohort study*—Summarise follow-up time (eg, average and total amount) | *n/a* |  |
| Outcome data | 15* | *Cohort study*—Report numbers of outcome events or summary measures over time | *n/a* |  |
|  |  | *Case-control study—*Report numbers in each exposure category, or summary measures of exposure | *n/a* |  |
|  |  | Cross-sectional study—Report numbers of outcome events or summary measures | 11&12 | Table 3 & 4 (Overall SBS) |
| Main results | 16 | (*a*) Give unadjusted estimates and, if applicable, confounder-adjusted estimates and their precision (eg, 95% confidence interval). Make clear which confounders were adjusted for and why they were included | 11 &12 | Use different precision method Table 3 |
|  |  | (*b*) Report category boundaries when continuous variables were categorized | n/a |  |
|  |  | (*c*) If relevant, consider translating estimates of relative risk into absolute risk for a meaningful time period | n/a | Use different metrices (comparing the algorithm) |

Continued on next page

| Other analyses | 17 | Report other analyses done—eg analyses of subgroups and interactions, and sensitivity analyses | 12&13 | Table 4, Table 5 and Table 6 |
| --- | --- | --- | --- | --- |
| Discussion | | | | |
| Key results | 18 | Summarise key results with reference to study objectives | 15 | The substantial size and suitability of our dataset, with a proportional 50% allocated for training and testing, aligns with epidemiological approaches for case-control study designs. This partitioning was vital to avoid artificially inflating odds ratios, which can occur if logistic regression is used for model training and evaluation |
| Limitations | 19 | Discuss limitations of the study, taking into account sources of potential bias or imprecision. Discuss both direction and magnitude of any potential bias | 16 | Specific chapter/ section on this matter |
| Interpretation | 20 | Give a cautious overall interpretation of results considering objectives, limitations, multiplicity of analyses, results from similar studies, and other relevant evidence | 16 |  |
| Generalisability | 21 | Discuss the generalisability (external validity) of the study results | 16 |  |
| Other information | |  | | |
| Funding | 22 | Give the source of funding and the role of the funders for the present study and, if applicable, for the original study on which the present article is based | n/a |  |

*Give information separately for cases and controls in case-control studies and, if applicable, for exposed and unexposed groups in cohort and cross-sectional studies.

**Note:** An Explanation and Elaboration article discusses each checklist item and gives methodological background and published examples of transparent reporting. The STROBE checklist is best used in conjunction with this article (freely available on the Web sites of PLoS Medicine at http://www.plosmedicine.org/, Annals of Internal Medicine at http://www.annals.org/, and Epidemiology at http://www.epidem.com/). Information on the STROBE Initiative is available at www.strobe-statement.org.
